# Incorporation of a Machine Learning Algorithm With Object Detection Within the Thyroid Imaging Reporting and Data System Improves the Diagnosis of Genetic Risk

**DOI:** 10.3389/fonc.2020.591846

**Published:** 2020-11-12

**Authors:** Shuo Wang, Jiajun Xu, Aylin Tahmasebi, Kelly Daniels, Ji-Bin Liu, Joseph Curry, Elizabeth Cottrill, Andrej Lyshchik, John R. Eisenbrey

**Affiliations:** ^1^ Department of Radiology, Thomas Jefferson University, Philadelphia, PA, United States; ^2^ Department of Ultrasound, Nanjing First Hospital, Nanjing Medical University, Nanjing, China; ^3^ Department of Otolaryngology, Thomas Jefferson University, Philadelphia, PA, United States; ^4^ Department of Otolaryngology, University of Pittsburgh Medical Center, Pittsburgh, PA, United States

**Keywords:** machine learning, thyroid nodules, next generation sequencing, object detection, Thyroid Imaging Reporting and Data System classification

## Abstract

**Background:**

The role of next generation sequencing (NGS) for identifying high risk mutations in thyroid nodules following fine needle aspiration (FNA) biopsy continues to grow. However, ultrasound diagnosis even using the American College of Radiology’s Thyroid Imaging Reporting and Data System (TI-RADS) has limited ability to stratify genetic risk. The purpose of this study was to incorporate an artificial intelligence (AI) algorithm of thyroid ultrasound with object detection within the TI-RADS scoring system to improve prediction of genetic risk in these nodules.

**Methods:**

Two hundred fifty-two nodules from 249 patients that underwent ultrasound imaging and ultrasound-guided FNA with NGS with or without resection were retrospectively selected for this study. A machine learning program (Google AutoML) was employed for both automated nodule identification and risk stratification. Two hundred one nodules were used for model training and 51 reserved for testing. Three blinded radiologists scored the images of the test set nodules using TI-RADS and assigned each nodule as high or low risk based on the presence of highly suspicious imaging features on TI-RADS (very hypoechoic, taller-than-wide, extra-thyroidal extension, punctate echogenic foci). Subsequently, the TI-RADS classification was modified to incorporate AI for T4 nodules while treating T1-3 as low risk and T5 as high risk. All diagnostic predictions were compared to the presence of a high-risk mutation and pathology when available.

**Results:**

The AI algorithm correctly located all nodules in the test dataset (100% object detection). The model predicted the malignancy risk with a sensitivity of 73.9%, specificity of 70.8%, positive predictive value (PPV) of 70.8%, negative predictive value (NPV) of 73.9% and accuracy of 72.4% during the testing. The radiologists performed with a sensitivity of 52.1 ± 4.4%, specificity of 65.2 ± 6.4%, PPV of 59.1 ± 3.5%, NPV of 58.7 ± 1.8%, and accuracy of 58.8 ± 2.5% when using TI-RADS and sensitivity of 53.6 ± 17.6% (p=0.87), specificity of 83.3 ± 7.2% (p=0.06), PPV of 75.7 ± 8.5% (p=0.13), NPV of 66.0 ± 8.8% (p=0.31), and accuracy of 68.7 ± 7.4% (p=0.21) when using AI-modified TI-RADS.

**Conclusions:**

Incorporation of AI into TI-RADS improved radiologist performance and showed better malignancy risk prediction than AI alone when classifying thyroid nodules. Employing AI in existing thyroid nodule classification systems may help more accurately identifying high-risk nodules.

## Introduction

In 2020, there are expected to be 52,890 new cases of thyroid cancer and 2,180 thyroid cancer related deaths in the United States alone (1). While relatively common (1.3% of people will be diagnosed with thyroid cancer at some point in their lifetime), overall survival is excellent, with a 5-year survival rate of 98.3% (1). However, the clinical burden of diagnosing and managing thyroid nodules continues to grow due to incidental findings on unrelated work-ups. Ultrasound is widely used as first-line imaging modality for the evaluation of thyroid nodules. The presence of high-risk features on thyroid ultrasound informs subsequent clinical decisions which aim to prevent missed diagnoses of thyroid cancers through selective biopsy while avoiding over-management of benign nodules.

The use of fine needle aspiration (FNA) biopsy, which enables cytology to be evaluated using the Bethesda System for Reporting Thyroid Cytopathology has reduced the number of diagnostic surgical thyroidectomies by half while doubling the number of identified thyroid cancers (2). However, 20-30% of all FNA samples fall into an indeterminate category such as follicular lesion of undetermined significance or atypia of undetermined significance (FLUS/AUS, Bethesda III) or suspicious for follicular neoplasm (Bethesda IV). The risk of malignancy with these cytologic classifications range from 6%–40% (3–6) and importantly, can only be evaluated following surgical resection. The Thyroid Imaging, Reporting and Data System (TI-RADS) was recently developed by the American College of Radiology (ACR) to standardize reporting of thyroid ultrasound exams, provide a system for differentiation of benign and malignant thyroid nodules and unify patient management recommendations (7). Recent reports have shown the use of these guidelines improves the classification of thyroid nodules (8–10). However, while this system provides reasonably high sensitivities in identifying thyroid carcinoma, it suffers from a poor overall specificity. Consequently, overtreatment of benign or indolent disease remains a significant clinical problem. Next-generation sequencing (NGS) identification of cancer-associated genes has improved risk stratification of indeterminate nodules. Thyroid cancer has specific genetic variations, such as point mutations of proto-oncogenes and chromosomal rearrangements that are related to histopathologic subtype and malignancy. NGS has been used for risk stratification of thyroid cancer based on the malignancy classification (4, 11–13). However, TI-RADS has been shown to perform poorly in Bethesda III-IV nodules undergoing NGS, with accuracies of 50%–75% (14–16). Additionally, it has been shown that inter-reader agreement using TI-RADS is relatively low in this population of nodules (14).

Numerous artificial intelligence (AI) models have been developed for use in thyroid ultrasound and have shown encouraging results in evaluating nodules and improving radiologic workflow (17–21). Work on thyroid nodule detection has also recently emerged and these tools are now becoming commercially available (22, 23). However, the vast majority of these studies focused on stratifying malignant from benign lesions in general population (containing an overwhelming large presence of benign nodules easily ruled out on ultrasound) or rely on surgical pathology following surgical excision which often omit or limit nodules falling into Bethesda III and IV categories on FNA. Diagnostic imaging and radiomic approaches are greatly needed to improve the management of these indeterminate nodules. Our group has recently explored the use of an ultrasound-based machine learning algorithm for genetic risk stratification of thyroid nodules, using NGS as a reference standard. Results of this study were encouraging, with specificity of 97% and overall accuracy of 77% (21). This model employed a Google AutoML algorithm (AutoML Vision; Google LLC), which benefits from cloud computing (thereby reducing localized hardware requirements) and transfer learning which dramatically reduces the data input requirements compared to conventional AI models (24, 25). The complete details of this proprietary algorithm are not provided, although it is stated it relies on neural architecture search approaches with reinforced learning using an internal validation dataset (24). Although we have been encouraged by our initial findings, we believe this algorithm’s “black box” approach and failure to incorporate into existing standardized scoring systems will limit clinical adoption. Consequently, the aim of this study was to explore the use of a machine leaning-based ultrasound AI model that provides object detection capabilities and to evaluate radiologist performance when this model is incorporated into the TI-RADS scoring system on a group of Bethesda III and IV “indeterminate” nodules. Object detection capabilities alert the reader to the area of the image being used for diagnosis, thus reducing the “black box” of unknown computations, which limited prior work by our group.

## Material and Methods

### Data Source and Extraction

This retrospective clinical study was approved by the Institutional Review Board (IRB) of Thomas Jefferson University Hospital. Informed consent was waived. Data were retrieved from department Picture Archiving and Communication System (PACS) and consisted of ultrasound images acquired at our institution immediately before or during FNA. The decision to perform FNA as part of the patient’s clinical care was based on the presence of known suspicious features on ultrasound (very hypoechoic, taller-than-wide, extra-thyroidal extension, punctate echogenic foci), TI-RADS classification, and patients’ risk factors. All of the above was reviewed with the patient who were presented with several options, including conservative management, imaging surveillance or to proceed with FNA. Inclusion criteria consisted of all patients who underwent thyroid ultrasound imaging and ultrasound-guided FNA with next-generation sequencing (NGS) with or without surgical pathology between January 2017 and August 2019. This relatively short eligibility window was based on availability of NGS, which was adopted for clinical care by our institution in January 2017. An institutional NGS panel of 23 evidence-based gene mutations associated with thyroid malignancy served as a reference to mark FNA samples as high- or low-risk. This 23-gene panel is summarized in **Table 1** and served as a rule-in test with samples containing one or more high-risk mutations being classified as high risk for malignancy, whereas samples with no mutation or a mutation considered to be of low or unknown risk were classified as low risk for malignancy by the molecular testing report. In cases where total thyroidectomy or lobectomy were performed following ultrasound imaging, subsequent malignant or benign pathology were treated as high or low risk respectively for the purpose of this study.

**Table 1:** High risk genes on NGS used as a reference standard.

Gene	Human Genome Region
AKT1	aa 17-18
APC	aa 178-291 and 312-1594 with splice sites
AXIN1	aa 1-688 and 731-865 with splice sites
BRAF	aa 594-606, 439-478
CDKN2A	Full with splice sites
CTNNB1	aa 6-60
DNMT3A	aa 881-883
EGFR	Exons 18,19,20,21
EIF1AX	aa 1-6, 35-86, and 115-147
GNAS	aa 201-203 and 226-227
HRAS	aa 10-14 and 60-62 and 146
IDH1	aa 67-71, 123-134
KRAS	aa 10-14 and 60-62 and 146
NDUFA13	Full with splice sites
NRAS	aa 10-14 and 60-62 and 146
PIK3CA	aa 520-554 and 980-1069
PTEN	Full with splice sites
RET	aa 883, 918, 588-636
SMAD4	aa 36-552 with splice sites
TERT	promoter chr5: 1295228 and 1295250
TP53	aa 26-393 with splice sites
TSHR	Full with splice sites
VHL	Full with splice sites

*aa denotes amino acid residue numbers.

### Data Formatting and Model Training

Two hundred forty-nine patients with Bethesda III-IV nodules were used for this study. Ultrasound images contained both the nodule in question and anterior neck muscles and were collected on a wide variety of ultrasound scanners as part of the patient’s clinical care. For image formatting, patient information, manufacturer label, and scale bars were removed *via* a cropping script written in Matlab (2016a, The Mathworks Inc., Natick, MA). A summary of the training and prediction dataset is show in **Table 2**. Eighty percent of the patients (198 patients with 201 total lesions) were used for model training. This training set consisted of 143 low-risk cases and 58 high-risk cases. In order to generate a large training dataset, all available B-mode images focused on the lesion were utilized to form the training dataset for the model, resulting in 488 low-risk and 228 high-risk images. Randomization was performed automatically by the algorithm, but there was no nodule overlap between training and testing sets. Restricting nodules to either test or training sets ensures that the algorithm does not have the advantage of being tested on an image from a nodule after being trained on images from the same nodule.

**Table 2:** Summary of training and prediction dataset composition.

Dataset/# of images	Low-risk	High-risk	Total
Training dataset	488	228	716
Prediction dataset	25	26	51

**Figure 1:**
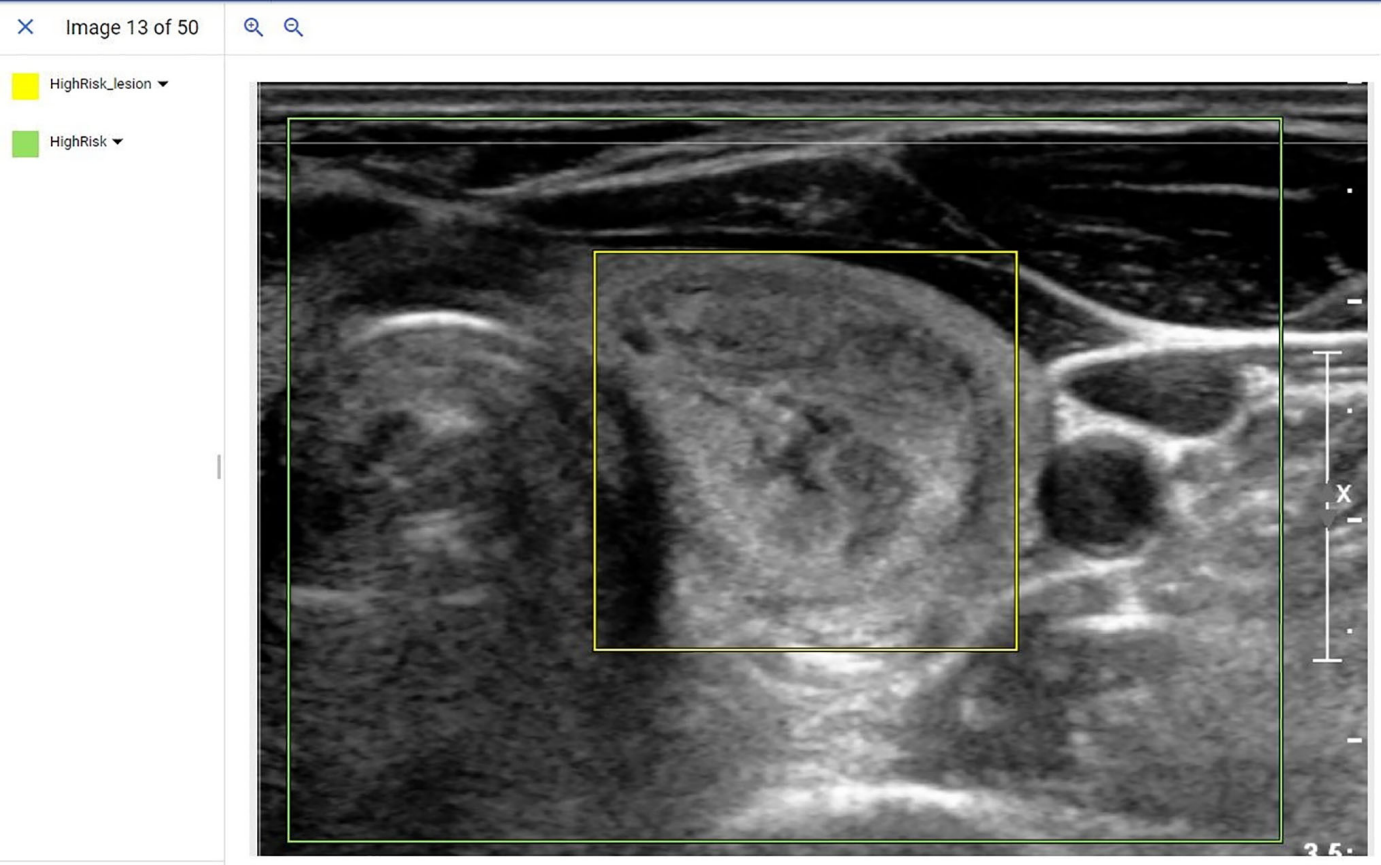
Example of a de-identified image being input into the AutoML object detection model. In this example, radiologists used bounding boxes to mark the location of the lesion (Yellow bounding box) and overall image (Green bounding box). The label was assigned with bounding boxes where the yellow bounding box indicated a high-risk lesion and the green bounding box indicated an ultrasound image that contained a high-risk lesion.

Following de-identification, training images were uploaded into the Google AutoML object detection model running on the cloud platform. A radiologist (JX) from a high volume academic medical centers with over 10 years of thyroid ultrasound experience and blinded to the NGS and pathology results used bounding boxes and labels to mark the location of the nodule in question as well as a cropped area of the larger image containing the lesion. An example of this cropped image and subsequent bounding boxes placed to train the location and genetic risk of the model is provided in **Figure 1**. Following data input, the model was trained unsupervised using a 16 nodal hour training condition, as the product documentation warns that additional nodal training hours may result in model overfitting.

### Evaluation of Model and Radiologist Performance

Twenty percent of cases (n=51) were randomly selected to evaluate the model’s performance [this split is recommended by the product literature and a commonly accepted ratio for validation of AI algorithms (24, 25)]. In this test, or more aptly named prediction dataset, there were 25 low-risk and 26 high-risk cases by either NGS or final pathology. One image was selected by a radiologist from each case to form the prediction dataset that resulted in 51 prediction images. Prediction images were uploaded into the pre-trained object detection model which in turn generated a processed image identifying the nodule, risk assignment, and overall confidence score (0-1) of the risk assignment. In order to draw decisions from the prediction results, a confidence score of 0.81 was utilized. This threshold was determined by the model training parameters as well as the threshold at which the most cases could be provided with an assigned risk by the model. Cases with a prediction certainty greater than 0.81 of either high- or low-risk were assigned to that diagnosis. Cases in which the nodule was not conclusively characterized included cases where the model provided a confidence ≤ 0.81 or in which the model provided both high- and low-risk labels to the same lesion with > 0.81 confidence. These AI findings were then considered non-applicable (NA).

For the initial readings, three blinded radiologists with over 10 years of experience in thyroid ultrasound (JX, J-BL, AL) individually scored each nodule using the ACR TI-RADS scoring system (7). The radiologists were also asked to classify lesions as high or low risk based on TI-RADS imaging characteristics of very hypoechoic, taller-than-wide, extra-thyroidal extension and punctate echogenic foci. These classifications were later used to compare radiologist performances with and without AI assistance. In the post-AI phase, nodules with a radiologist-assigned TR1-3 were assigned as low risk, radiologist-assigned TR4s were classified using the AI model, and TR5 was considered high risk. These assignments were based on the defined risk assessments within the TI-RADS system (Benign to Mildly Suspicious, Moderately Suspicious, Highly Suspicious).

All reads and predictions were compared to NGS or final surgical pathology (when available) as a reference standard. Statistical analysis was performed in GraphPad Prism Version 8.4.2 for Windows, GraphPad Software, La Jolla California USA. Data was presented as mean ± standard deviation. A paired t-test was employed to compare the radiologist performances in pre- and post- incorporation of AI into the TI-RADS grading system.

## Results

The 249 patients included 53 males and 196 females with an average age of 56 ± 14 years old, and an average lesion dimension of 2.8 ± 1.4 cm. Each case from the 249 patients included multiple images that represented the sagittal and transverse views of the nodule, providing a total of 716 images. Both surgical pathology and NGS were available in 59 patients. Within the subset of indeterminate (Bethesda III and IV nodules) that later underwent surgery, NGS provided a sensitivity of 71.4%, specificity of 58.6%, a positive predictive value (PPV) of 60.6%, negative predictive value (NPV) of 69.2% and an accuracy of 64.4%.

**Figure 2:**
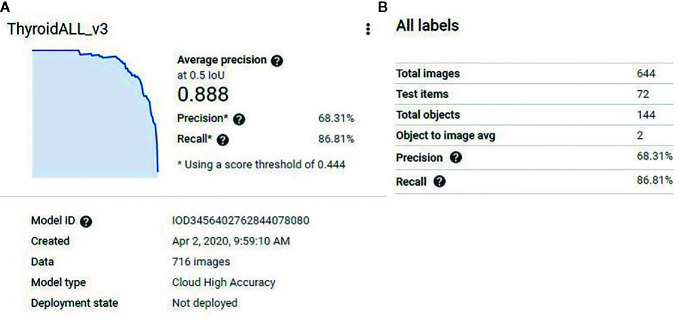
**(A)** The model performance report generated by the platform. The model had an AUC of 0.889. **(B)** The model split the 716 training images into new training (644 images) and new testing (72) datasets. At a confidence score level of 0.44, the model had 68.31% precision and 86.81% recall for the new testing (72 images) dataset.

The function of the object detection model was to detect all predetermined objects (in this study, the nodules themselves) in a given image and provide confidence scores (certainty) for the objects it detected. After the model training was finished, a model performance report was generated by the platform (**Figure 2A**). The model split the 716 images into new training (644 images) and internal testing (72 images) datasets (**Figure 2B**). From the report, based on the 72 internal validation images, the model had an AUC of 0.889 with a precision of 68.31%, and a recall of 86.81% at a confidence score level of 0.44.

**Figure 3:**
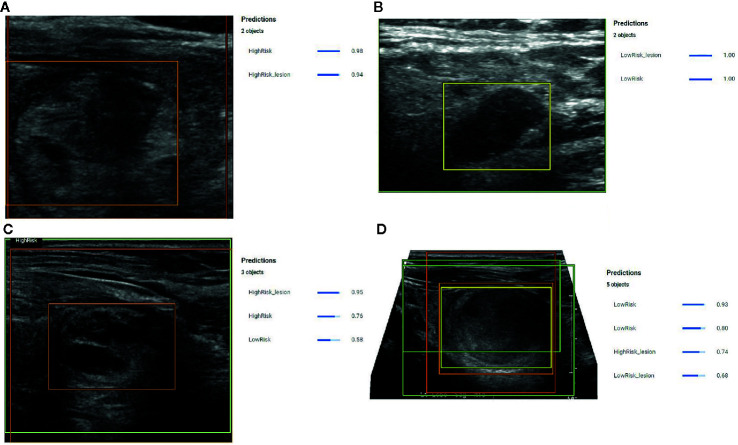
**(A)** Example prediction from the Object Detection Model that correctly detected a nodule and correctly assigned a high-risk label with 98% certainty. The position of the high-risk nodule was marked by the orange color bounding box drawn by the model. **(B)** Example prediction from the Object Detection Model that correctly detected the nodule and correctly designated the lesion as low risk with 100% confidence. The position of the low-risk nodule was marked by the yellow bounding box drawn by the model. **(C)** Example prediction from the Object Detection Model that detected a high-risk lesion, a high-risk area, and a low-risk area with confidence scores of 0.96, 0.76, and 0.56, respectively. The position of the high-risk nodule was marked with the orange bounding box drawn by the model. **(D)** Example prediction from the Object Detection Model that detected the nodule but assigned both high-risk and low-risk labels. The model provided a confidence score of 0.74 for the nodule to be high risk and a confidence score of 0.68 for the nodule to be low risk. The model also indicated two low risk areas with a confidence score of 0.93 and 0.8.

When this model was applied to the 51 prediction images (with no images from these patients used during model development) it correctly identified the nodule in all 51 (100%) cases. Correct identification in all cases was confirmed by a blinded radiologist and each image contained only 1 nodule. Following identification, the risk prediction portion of the model showed three distinct behaviors. In the first behavior, the model detected the nodules and provided a confidence score with greater than 80% certainty (**Figures 3A, B** **)**, indicating a reliable interpretation. In the second behavior, the model correctly identified the location of the nodule, but classified it as both a high-risk and low-risk area with different confidence levels (**Figure 3C**), thereby making the reliability of the diagnosis dependent on the confidence threshold. In the final behavior, the model detected nodules, but assigned both high-risk and low-risk labels to the same nodule with different confidence scores (**Figure 3D**), thereby indicating an unreliable diagnosis.

**Figure 4:**
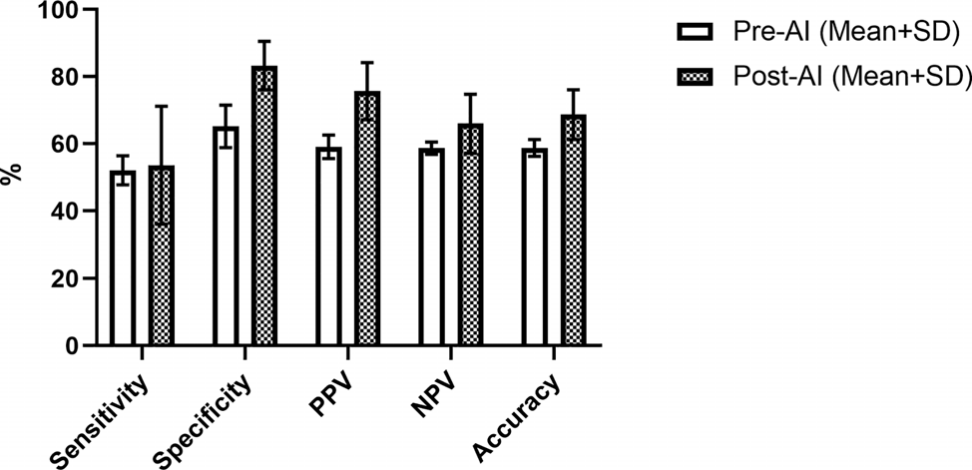
Radiologist performance using TI-RADS criteria alone (white) and TI-RADS with AI assistance (checkered) for predicting the risk of thyroid nodules on B-mode ultrasound.

The overall performance of the model in the prediction test set is summarized by the confusion matrix in **Table 3**. Final diagnosis was provided in 47 of 51 cases based on the confidence score criteria described above. The corresponding sensitivity, specificity, PPV, NPV, and accuracy are provided in **Table 4** as well as the radiologist performance post- incorporation of the AI results into the TI-RADS scoring system. As a stand-alone model, the AI platform demonstrated a sensitivity of 73.9%, specificity of 70.8%, PPV of 70.8%, NPV of 73.9%, and overall accuracy of 72.4%. The performance of the AI model was better although not statistically significant than the performance of three experienced radiologists when using TI-RADS classification alone to identify suspicious imaging features (sensitivity of 52.1 ± 4.4%, specificity of 65.2 ± 6.4%, PPV of 59.1 ± 3.5%, NPV of 58.7 ± 1.8%, and accuracy of 58.8 ± 2.5%). Importantly, AI-modified TI-RADS radiologist improved the diagnostic performance in these nodules (sensitivity of 53.6 ± 17.6%, specificity of 83.3 ± 7.2%, PPV of 75.7 ± 8.5%, NPV of 66.0 ± 8.8%, and accuracy of 68.7 ± 7.4%). While not statistically significant (p > 0.06), all radiologist performance metrics improved with the incorporation of AI (**Figure 4**).

**Table 3:** Confusion matrix for the prediction images with AI diagnosis.

	High-Risk	Low-Risk
Prediction High-Risk	17	7
Prediction Low-Risk	6	17

**Table 4:** Sensitivity, specificity, PPV, and NPV, and accuracy from the AI alone, TI-RADS alone, and AI + TI-RADS.

	Sensitivity (95% CI)	Specificity (95% CI)	PPV (95% CI)	NPV (95% CI)	Accuracy (95% CI)
Object Detection Model	73.9% (51.6–90.0)	70.8% (48.9–87.4)	70.8% (55.4–82.6)	73.9% (57.5–85.5)	72.4% (57.4–84.4)
Radiologist Alone	52.1 ± 4.4% (30.6–73.1)	65.2 ± 6.4% (43.4–83.2)	59.1 ± 3.5% (42.4–74.0)	58.7 ± 1.8% (45.9–70.5)	58.8 ± 2.5% (43.6–73.0)
Radiologist Post-AI	53.6 ± 17.6% (32.7–73.6)	83.3 ± 7.2% (62.9–94.9)	75.7 ± 8.5% (53.2–89.0)	66.0 ± 8.8% (54.6–75.9)	68.7 ± 7.4% (53.8–81.4)
	p = 0.87	p = 0.06	p = 0.13	p = 0.31	p = 0.21

95% Confidence interval: 95% CI.

## Discussion

Overall, the object detection model used in this study showed promising performance. The model correctly designated the location of all 51 thyroid nodules in the testing set. Although four cases (7.8% of the total) needed to be removed due to conflicting predictions, when predicting high vs low risk for malignancy in the identified nodules, the AI model achieved reasonably high diagnostic performance during testing (**Table 4**). Notably, these values are higher than the performance of NGS in the subset of patients with both NGS and pathology available, although the model itself was built primarily on NGS data. These challenges highlight the need for improved diagnostic tools for characterizing nodules that meet radiographic criteria for biopsy but have indeterminate cytopathology following FNA.

Most metrics improved with the combination of radiologists with AI vs. AI alone. While not statistically significant, these improvements are promising given the low diagnostic performance of TI-RADS for predicting genetic risk we have previously reported in a significantly larger sample size (14). These improvements appear to rely on clusters of atypical cases falling in the moderately suspicious category (TR4) on the TI-RADS system. For example, there were six nodules in the prediction set that all radiologists classified as low risk, but which fell into high risk categories based on NGS or final pathology and two nodules that all radiologists identified as high-risk but were low risk on NGS. The algorithm correctly classified both of these low-risk nodules and four of six of the high-risk nodules. From a clinical perspective, our data suggests incorporation of the AI model would allow more patients with low risk nodules to potentially forgo surgical intervention while also improving the identification of higher-risk nodules in need of further intervention. These results are highly encouraging given the nodules used in this model all met radiographic criteria for biopsy and had indeterminate cytopathology requiring further risk stratification with NGS testing to help in risk assessment.

While many AI models have been published for the diagnosis and management of thyroid nodules, relatively few have focused on genetic risk of nodules referred for FNA. A deep learning algorithm described in the Buda et al. study achieved a higher sensitivity (87%) and specificity (52%) than the mean sensitivity (83%) and specificity (48%) of nine radiologists for thyroid nodule biopsy referral based on ultrasound images (26). Wildman-Tobriner et al. modified the ACR TI-RADS by a genetic machine learning algorithm and called it AI-TIRADS. Their proposed AI-TIRADS system reassigned point values based on AI interpretation, assigning 1 point for taller than wide shape, 2 points for hypoechogenicity, irregular and lobulated margins, peripheral echogenic foci and 3 points for solid composition, very hypoechoic lesions, extra thyroidal extensions, and punctuate echogenic foci. The rest of ACR-TIRADS features received zero points. Similar to our study their results improved specificity greatly when compared to ACR TI-RADS scoring alone (27).

Incorporation of radiology AI into established classification systems is likely to aid in clinical adoption. Results from this study demonstrate the potential improvements of combining both advanced machine learning programs with experienced radiologists in a particularly challenging nodule population. Similarly, a need to explain and validate an AI model’s decision making is likely to be vital for clinical adoption. Most of the AI algorithms applied for the classification of thyroid nodules do not provide an indication of what part of the image is being used for diagnosis and serve as a black box. Additionally, the algorithm performance can be affected by the training node hours (computation time allotted during training), noise and contrast level of images, and parameter sampling. The model employed in this study is still subject to some of these limitations. However, the incorporation of the object detection model and confidence scoring provides reassurance that the correct area is being evaluated and multiple diagnostic criteria are being weighed.

While this study has shown encouraging results, limitations exist and should be addressed moving forward. Due to its retrospective, single center design, the study was limited in sample size and did not utilize a standardized imaging protocol. Larger, multi-center prospective trials are needed to fully validate findings. While the algorithm’s detection of the lesion agreed with blinded radiologists in 100% of cases, the program’s use of a rectangular bounding box prohibits traditional object detection quantitative measures such as intersection over union (28). Similarly, while automated identification of the nodule reassures the reader the correct area is being used during prediction, it is possible areas outside the nodule but within the rectangular bounding box are being used for forecasting genetic risk. Future work should explore feature extraction to confirm the appropriate region within the bounding box is being used while also identifying key features being used by the algorithm (thereby also reducing the ‘black box’ nature to some degree). Our reference standard used primarily NGS but was supplemented by some cases with final pathology following surgical resection to meet the data requirements of the high-risk label. While the goal of this study was to predict genetic risk, ideally longer-term surveillance should be incorporated to correlate genetic risk with clinical outcomes. Lastly, for the consistency of the comparison we showed only one image per nodule to clinicians while in clinical practice they usually assess the nodule risk by looking at multiple images of the nodule in different planes.

Despite these limitations, initial results have been encouraging and may serve as a roadmap for the incorporation of ultrasound-AI into clinical practice. Ultimately, we expect these approaches to help guide radiologist decision making by providing management direction in nodules in which the decision to perform FNA is unclear based on existing risk classification systems. Similarly, findings from Bethesda III and IV nodules may be combined with known risk factors and patient preference to help guide the selection of either active surveillance or surgical intervention.

## Conclusion

The incorporation of AI algorithms into daily clinical practice can potentially assist radiologists in decision making and act as an auxiliary tool. In this study, AI-modified TI-RADS improved the performance of both radiologists and AI alone when classifying the genetic risk of thyroid nodules for further management while looking specifically at a subset of nodules which were indeterminate (Bethesda III/IV) on cytopathology. Clinically, this approach suggests the ability to more accurately identify truly high-risk nodules on initial ultrasound and prevent invasive interventions of lower-risk nodules.

## Data Availability Statement

The raw data supporting the conclusions of this article will be made available by the authors, without undue reservation.

## Ethics Statement

The studies involving human participants were reviewed and approved by Thomas Jefferson University IRB. Written informed consent for participation was not required for this study in accordance with the national legislation and the institutional requirements.

## Author Contributions

SW—Study design, data processing, data analysis, manuscript writing, manuscript editing. JX—Study design, data processing, data analysis, manuscript writing, manuscript editing. AT—Data processing, data analysis, manuscript editing. KD—Data processing, data analysis, manuscript writing, manuscript editing. J-BL—Data analysis, manuscript writing, manuscript editing. JC—Study conceptualization, data analysis, manuscript editing. EC—Study conceptualization, data analysis, manuscript writing, manuscript editing. AL—Study conceptualization, data analysis, manuscript writing, manuscript editing. JE—Study conceptualization, study design, data analysis, manuscript writing, manuscript editing, institutional approval. All authors contributed to the article and approved the submitted version.

## Conflict of Interest

The authors declare that the research was conducted in the absence of any commercial or financial relationships that could be construed as a potential conflict of interest.

## References

[1] National Cancer Institute, Surveillance, Epidemiology and End Results Program Cancer Stat Facts: Thyroid Cancer. Available at: www.seer.cancer.gov/statfacts/html/thyro.html (Accessed April 15, 2020).

[2] Gharib H Goellner JR Fine-needle aspiration biopsy of the thyroid: an appraisal. Ann Intern Med (1993) 118:282–9. 10.7326/0003-4819-118-4-199302150-00007 8420446

[3] Singh Ospina N Iniguez-Ariza NM Castro MR Thyroid nodules: diagnostic evaluation based on thyroid cancer risk assessment. BMJ (2020) 368:l6670. 10.1136/bmj.l6670 31911452

[4] Cha YJ Koo JS Next-generation sequencing in thyroid cancer. J Transl Med (2016) 14:322. 10.1186/s12967-016-1074-7 27871285 PMC5117557

[5] Sahli ZT Smith PW Umbricht CB Zeiger MA Preoperative Molecular Markers in Thyroid Nodules. Front Endocrinol (Lausanne) (2018) 9:179. 10.3389/fendo.2018.00179 29720964 PMC5915469

[6] Cibas ES Ali SZ The 2017 Bethesda System for Reporting Thyroid Cytopathology. Thyroid (2017) 27:1341–6. 10.1089/thy.2017.0500 29091573

[7] Tessler FN Middleton WD Grant EG Hoang JK Berland LL Teefey SA ACR Thyroid Imaging, Reporting and Data System (TI-RADS): White Paper of the ACR TI-RADS Committee. J Am Coll Radiol (2017) 14:587–95. 10.1016/j.jacr.2017.01.046 28372962

[8] Ahmadi S Herbst R Oyekunle T Jiang X Strickland K Roman S Using the ATA and ACR Ti-Rads Sonographic Classifications as Adjunctive Predictors of Malignancy for Indeterminate Thyroid Nodules. Endocr Pract (2019) 25:908–17. 10.4158/EP-2018-0559 31170369

[9] Lauria Pantano A Maddaloni E Briganti SI Beretta Anguissola G Perrella E Taffon C Differences between ATA, AACE/ACE/AME and ACR TI-RADS ultrasound classifications performance in identifying cytological high-risk thyroid nodules. Eur J Endocrinol (2018) 178:595–603. 10.1530/EJE-18-0083 29626008

[10] Macedo BM Izquierdo RF Golbert L Meyer ELS Reliability of Thyroid Imaging Reporting and Data System (TI-RADS), and ultrasonographic classification of the American Thyroid Association (ATA) in differentiating benign from malignant thyroid nodules. Arch Endocrinol Metab (2018) 62:131–8. 10.20945/2359-3997000000018 PMC10118978 29641731

[11] Steward DL Carty SE Sippel RS Yang SP Sosa JA Sipos JA Performance of a Multigene Genomic Classifier in Thyroid Nodules With Indeterminate Cytology: A Prospective Blinded Multicenter Study. JAMA Oncol (2019) 5:204–12. 10.1001/jamaoncol.2018.4616 PMC6439562 30419129

[12] Bandoh N Akahane T Goto T Kono M Ichikawa H Sawada T Targeted next-generation sequencing of cancer-related genes in thyroid carcinoma: A single institution’s experience. Oncol Lett (2018) 16:7278–86. 10.3892/ol.2018.9538 PMC6256352 30546467

[13] Nishino M Molecular cytopathology for thyroid nodules: A review of methodology and test performance. Cancer Cytopathol (2016) 124:14–27. 10.1002/cncy.21612 26348024

[14] Daniels KE Xu J Liu JB Chen X Huang J Patel J Diagnostic value of TI-RADS classification system and next generation genetic sequencing in indeterminate thyroid nodules. Acad Radiol (2020) S1076-6332(20):30460–8. 10.1016/j.acra.2020.07.037 32839097

[15] Chaigneau E Russ G Royer B Bigorgne C Bienvenu-Perrard M Rouxel A TIRADS score is of limited clinical value for risk stratification of indeterminate cytological results. Eur J Endocrinol (2018) 179:13–20. 10.1530/EJE-18-0078 29703794

[16] Ahmadi S Herbst R Oyekunle T Jian X Strickland K Roman S Using the ATA and ACR TI-RADS sonographic classifications as adjunctive predictors of malignancy for indeterminate thyroid nodules. Endocr Pract (2019) 25(9):908–17. 10.4158/EP-2018-0559 31170369

[17] Ko SY Lee JH Yoon JH Na H Hong E Han K Deep convolutional neural network for the diagnosis of thyroid nodules on ultrasound. Head Neck (2019) 41:885–91. 10.1002/hed.25415 30715773

[18] Choi YJ Baek JH Park HS Shim WH Kim TY Shong YK A Computer-Aided Diagnosis System Using Artificial Intelligence for the Diagnosis and Characterization of Thyroid Nodules on Ultrasound: Initial Clinical Assessment. Thyroid (2017) 27:546–52. 10.1089/thy.2016.0372 28071987

[19] Thomas J Haertling T AIBx, Artificial Intelligence Model to Risk Stratify Thyroid Nodules. Thyroid (2020) 30(6):878–84. 10.1089/thy.2019.0752 32013775

[20] Zhang S Du H Jin Z Zhu Y Zhang Y Xie F A Novel Interpretable Computer-Aided Diagnosis System of Thyroid Nodules on Ultrasound Based on Clinical Experience. IEEE Access (2020) 8:53223–31. 10.1109/ACCESS.2020.2976495

[21] Daniels K Gummadi S Zhu Z Wang S Patel J Swendseid B Machine Learning by Ultrasonography for Genetic Risk Stratification of Thyroid Nodules. JAMA Otolaryngol Head Neck Surg (2019) 146(1):1–6. 10.1001/jamaoto.2019.3073 PMC6813575 31647509

[22] Wang L Yang S Yang S Zhao C Tian G Gao Y Automatic thyroid nodule recognition and diagnosis in ultrasound imaging with the YOLOvs neural network. World J Surg Oncol (2019) 17(1):12. 10.1186/s12957-019-1558-z 30621704 PMC6325802

[23] Abdolali F Kapur J Jaremko JL Noga M Hareendranathan AR Punithakumar K. Automated thyroid nodule detection from ultrasound imaging using deep convolutional neural networks. Comput Biol Med (2020) 122:103871. 10.1016/j.compbiomed.2020.103871 32658741

[24] Li FF Li J Cloud AutoML: Making AI accessible to every business. Google Cloud (2018). Available at: www.blog.google/products/google-cloud/cloud-automl-making-ai-accessible-every-business/ (Accessed April 27, 2020).

[25] Shuo W Liu JB Zhu Z Eisenbrey J Artificial Intelligence in Ultrasound Imaging: Current Research and Applications. Adv Ultrasound Diagn Ther (2019) 03:053–61. 10.37015/AUDT.2019.190811

[26] Buda M Wildman-Tobriner B Hoang JK Thayer D Tessler FN Middleton WD Management of Thyroid Nodules Seen on US Images: Deep Learning May Match Performance of Radiologists. Radiology (2019) 292:695–701. 10.1148/radiol.2019181343 31287391 PMC10257128

[27] Wildman-Tobriner B Buda M Hoang JK Middleton WD Thayer D Short RG Using Artificial Intelligence to Revise ACR TI-RADS Risk Stratification of Thyroid Nodules: Diagnostic Accuracy and Utility. Radiology (2019) 292:112–9. 10.1148/radiol.2019182128 31112088

[28] Handelman GS Kok HK Chandra RV Razavi AH Huang S Brooks M Peering into the black box of artificial intelligence: evaluation metrics of machine learning methods. AJR Am J Roentgenol (2019) 212(1):38–43. 10.2214/AJR.18.20224 30332290

